# *N*-(4-Chloro-3-nitro­phen­yl)succinamic acid

**DOI:** 10.1107/S1600536812008720

**Published:** 2012-03-03

**Authors:** U. Chaithanya, Sabine Foro, B. Thimme Gowda

**Affiliations:** a Department of Chemistry, Mangalore University, Mangalagangotri 574 199, Mangalore, India; b Institute of Materials Science, Darmstadt University of Technology, Petersenstrasse 23, D-64287 Darmstadt, Germany

## Abstract

In the title compound, C_10_H_9_ClN_2_O_5_, the nitro group is significantly twisted out of the plane of the benzene ring to which it is attached [dihedral angle = 27.4 (6)°]. In the crystal, mol­ecules are linked into centrosymmetric dimers *via* pairs of O—H⋯O hydrogen bonds. These dimers are further linked by N—H⋯O hydrogen bonds into double chains running along the *a* axis.

## Related literature

For our studies on the effects of substituents on the structures and other aspects of *N*-(ar­yl)-amides, see: Gowda *et al.* (2000 ▶); Chaithanya *et al.* (2012 ▶), on *N*-(ar­yl)-methane­sulfonamides, see: Gowda *et al.* (2007 ▶), on *N*-chloro­aryl­amides, see: Gowda *et al.* (2003 ▶); Jyothi & Gowda (2004 ▶) and on *N*-bromo­aryl­sulfonamides, see: Usha & Gowda (2006 ▶).
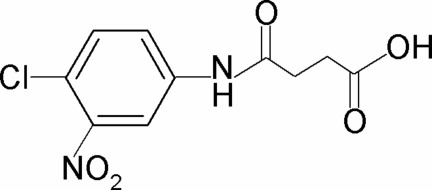


## Experimental

### 

#### Crystal data

C_10_H_9_ClN_2_O_5_
*M* *_r_* = 272.64
Monoclinic, 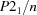

*a* = 4.8089 (8) Å
*b* = 10.278 (1) Å
*c* = 23.062 (3) Å
β = 90.69 (2)°
*V* = 1139.8 (3) Å^3^
*Z* = 4
Mo *K*α radiation
μ = 0.35 mm^−1^
*T* = 293 K
0.44 × 0.12 × 0.10 mm

#### Data collection

Oxford Diffraction Xcalibur diffractometer with a Sapphire CCD detector
Absorption correction: multi-scan (*CrysAlis RED*; Oxford Diffraction, 2009 ▶) *T*_min_ = 0.861, *T*_max_ = 0.966
4342 measured reflections
2305 independent reflections
1601 reflections with *I* > 2σ(*I*)
*R*_int_ = 0.015

#### Refinement

*R*[*F* ^2^ > 2σ(*F* ^2^)] = 0.065
*wR*(*F* ^2^) = 0.169
*S* = 1.05
2305 reflections
169 parameters
2 restraints
H atoms treated by a mixture of independent and constrained refinement
Δρ_max_ = 0.42 e Å^−3^
Δρ_min_ = −0.41 e Å^−3^

### 

Data collection: *CrysAlis CCD* (Oxford Diffraction, 2009 ▶); cell refinement: *CrysAlis RED* (Oxford Diffraction, 2009 ▶); data reduction: *CrysAlis RED*; program(s) used to solve structure: *SHELXS97* (Sheldrick, 2008 ▶); program(s) used to refine structure: *SHELXL97* (Sheldrick, 2008 ▶); molecular graphics: *PLATON* (Spek, 2009 ▶); software used to prepare material for publication: *SHELXL97*.

## Supplementary Material

Crystal structure: contains datablock(s) I, global. DOI: 10.1107/S1600536812008720/bt5832sup1.cif

Structure factors: contains datablock(s) I. DOI: 10.1107/S1600536812008720/bt5832Isup2.hkl

Supplementary material file. DOI: 10.1107/S1600536812008720/bt5832Isup3.cml

Additional supplementary materials:  crystallographic information; 3D view; checkCIF report

## Figures and Tables

**Table 1:** Hydrogen-bond geometry (Å, °)

*D*—H⋯*A*	*D*—H	H⋯*A*	*D*⋯*A*	*D*—H⋯*A*
N1—H1*N*⋯O1^i^	0.85 (2)	2.28 (3)	3.006 (3)	144 (3)
O3—H3*O*⋯O2^ii^	0.83 (2)	1.84 (2)	2.667 (3)	176 (4)

Symmetry codes: (i) 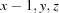; (ii) 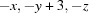.

**Fig. 1.**
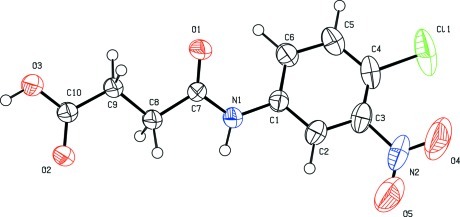
Molecular structure of the title compound, showing the atom labelling scheme and with displacement ellipsoids drawn at the 50% probability level.

**Fig. 2.**
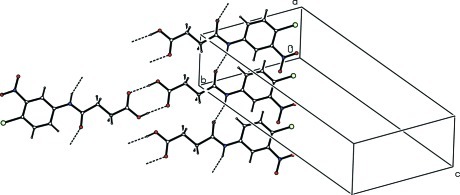
Molecular packing of the title compound with hydrogen bonding shown as dashed lines.

## supplementary crystallographic information

### Comment

As part of our studies on the substituent effects on the structures and other aspects of *N*-(aryl)-amides (Gowda *et al.*, 2000; Chaithanya *et al.*, 2012), *N*-(aryl)-methanesulfonamides (Gowda *et al.*, 2007); *N*-chloroarylsulfonamides (Gowda *et al.*, 2003; Jyothi & Gowda, 2004) and *N*-bromoaryl- sulfonamides (Usha & Gowda, 2006), in the present work, the crystal structure of *N*-(4-Chloro-3-nitrophenyl)succinamic acid has been determined (Fig. 1). The conformations of the N—H and the C=O bonds in the amide segment are *anti* to each other. But the N—H bond is *syn* to the *meta*–nitro group. The conformations of the amide C═O and the carboxyl C═O of the acid segment are *anti* to each other and both are *anti* to the H atoms on the adjacent –CH_2_ groups. Furthermore, the C═O and O—H bonds of the acid group are in *syn* position to each other, in contrast to the *anti* positions observed in *N*-(4-Chloro-3-nitro- phenyl)maleamic acid (I) (Chaithanya *et al.*, 2012).

The dihedral angle between the phenyl ring and the amide group in the title compound is 31.8 (2)°, compared to the value of 11.5 (3)° in (I).

In the structure, the O—H···O and N—H···O intermolecular hydrogen bonds link the molecules into double chains running along the a axis (Table 1, Fig. 2).

### Experimental

Succinic anhydride (0.025 mol) in toluene (25 ml) was treated dropwise with 4-chloro-3-nitroaniline (0.025 mol) also in toluene (20 ml) with constant stirring. The resulting mixture was stirred for about 30 min and set aside for an additional 30 min at room temperature for the completion of reaction. The mixture was then treated with dilute hydrochloric acid to remove the unreacted 4-chloro-3-nitroaniline. The resultant solid *N*-(4-Chloro-3-nitrophenyl)succinamic acid was filtered under suction and washed thoroughly with water to remove the unreacted succinic anhydride and succinic acid. It was recrystallized to constant melting point from ethanol. The purity of the compound was checked and characterized by its infrared spectra.

Rod like colorless single crystals of the title compound used in X-ray diffraction studies were grown in an ethanol solution by slow evaporation of the solvent (0.5 g in about 30 ml of ethanol) at room temperature.

### Refinement

All H atoms were located in a difference map Those bonded to C H atoms were positioned with idealized geometry using a riding model with the aromatic C—H = 0.93 Å and methylene C—H = 0.97 Å. The coordinates of the H atoms bonded to N and O were refined with the N—H and O—H distance restrained to 0.86 (2) Å and 0.82 (2)Å, respectively. All H atoms were refined with isotropic displacement parameters set at 1.2 *U*_eq_ of the parent atom.

### Crystal data

**Table:**

C_10_H_9_ClN_2_O_5_	*F*(000) = 560
*M**_r_* = 272.64	*D*_x_ = 1.589 Mg m^−^^3^
Monoclinic, *P*2_1_/*n*	Mo *K*α radiation, λ = 0.71073 Å
Hall symbol: -P 2yn	Cell parameters from 1280 reflections
*a* = 4.8089 (8) Å	θ = 2.6–27.7°
*b* = 10.278 (1) Å	µ = 0.35 mm^−^^1^
*c* = 23.062 (3) Å	*T* = 293 K
β = 90.69 (2)°	Rod, colourless
*V* = 1139.8 (3) Å^3^	0.44 × 0.12 × 0.10 mm
*Z* = 4	

### Data collection

**Table:**

Oxford Diffraction Xcalibur diffractometer with a Sapphire CCD detector	2305 independent reflections
Radiation source: fine-focus sealed tube	1601 reflections with *I* > 2σ(*I*)
Graphite monochromator	*R*_int_ = 0.015
Rotation method data acquisition using ω and phi scans	θ_max_ = 26.4°, θ_min_ = 2.7°
Absorption correction: multi-scan (*CrysAlis RED*; Oxford Diffraction, 2009)	*h* = −3→6
*T*_min_ = 0.861, *T*_max_ = 0.966	*k* = −12→11
4342 measured reflections	*l* = −28→26

### Refinement

**Table:**

Refinement on *F*^2^	Primary atom site location: structure-invariant direct methods
Least-squares matrix: full	Secondary atom site location: difference Fourier map
*R*[*F*^2^ > 2σ(*F*^2^)] = 0.065	Hydrogen site location: inferred from neighbouring sites
*wR*(*F*^2^) = 0.169	H atoms treated by a mixture of independent and constrained refinement
*S* = 1.05	*w* = 1/[σ^2^(*F*_o_^2^) + (0.0627*P*)^2^ + 1.4622*P*] where *P* = (*F*_o_^2^ + 2*F*_c_^2^)/3
2305 reflections	(Δ/σ)_max_ = 0.011
169 parameters	Δρ_max_ = 0.42 e Å^−^^3^
2 restraints	Δρ_min_ = −0.41 e Å^−^^3^

### Special details

**Table:**

Experimental. CrysAlis RED (Oxford Diffraction, 2009) Empirical absorption correction using spherical harmonics, implemented in SCALE3 ABSPACK scaling algorithm.
Geometry. All e.s.d.'s (except the e.s.d. in the dihedral angle between two l.s. planes) are estimated using the full covariance matrix. The cell e.s.d.'s are taken into account individually in the estimation of e.s.d.'s in distances, angles and torsion angles; correlations between e.s.d.'s in cell parameters are only used when they are defined by crystal symmetry. An approximate (isotropic) treatment of cell e.s.d.'s is used for estimating e.s.d.'s involving l.s. planes.
Refinement. Refinement of *F*^2^ against ALL reflections. The weighted *R*-factor *wR* and goodness of fit *S* are based on *F*^2^, conventional *R*-factors *R* are based on *F*, with *F* set to zero for negative *F*^2^. The threshold expression of *F*^2^ > σ(*F*^2^) is used only for calculating *R*-factors(gt) *etc*. and is not relevant to the choice of reflections for refinement. *R*-factors based on *F*^2^ are statistically about twice as large as those based on *F*, and *R*- factors based on ALL data will be even larger.

### Fractional atomic coordinates and isotropic or equivalent isotropic displacement parameters (Å^2^)

**Table:**

	*x*	*y*	*z*	*U*_iso_*/*U*_eq_	
C1	0.0737 (6)	0.7722 (3)	0.13397 (13)	0.0366 (7)	
C2	−0.0598 (7)	0.7250 (3)	0.18230 (14)	0.0438 (8)	
H2	−0.1913	0.7759	0.2009	0.053*	
C3	0.0015 (8)	0.6024 (3)	0.20300 (14)	0.0503 (9)	
C4	0.1914 (9)	0.5237 (3)	0.17540 (17)	0.0574 (10)	
C5	0.3217 (8)	0.5714 (3)	0.12722 (17)	0.0556 (9)	
H5	0.4498	0.5194	0.1082	0.067*	
C6	0.2671 (7)	0.6949 (3)	0.10641 (14)	0.0451 (8)	
H6	0.3597	0.7258	0.0740	0.054*	
C7	0.1739 (6)	0.9830 (3)	0.08795 (13)	0.0355 (7)	
C8	0.0419 (6)	1.1125 (3)	0.07321 (15)	0.0430 (8)	
H8A	−0.1226	1.0980	0.0493	0.052*	
H8B	−0.0156	1.1550	0.1087	0.052*	
C9	0.2393 (6)	1.1998 (3)	0.04144 (15)	0.0433 (8)	
H9A	0.4022	1.2140	0.0659	0.052*	
H9B	0.3000	1.1551	0.0068	0.052*	
C10	0.1234 (6)	1.3291 (3)	0.02419 (14)	0.0389 (7)	
N1	0.0022 (5)	0.8974 (3)	0.11358 (12)	0.0427 (7)	
H1N	−0.163 (4)	0.922 (3)	0.1198 (15)	0.051*	
N2	−0.1337 (9)	0.5654 (4)	0.25738 (15)	0.0712 (11)	
O1	0.4145 (4)	0.9578 (2)	0.07676 (12)	0.0550 (7)	
O2	−0.1033 (5)	1.3682 (2)	0.04216 (12)	0.0557 (7)	
O3	0.2758 (5)	1.3960 (2)	−0.00944 (13)	0.0588 (7)	
H3O	0.219 (8)	1.470 (2)	−0.0181 (18)	0.071*	
O4	−0.0219 (9)	0.4891 (5)	0.28923 (18)	0.1385 (19)	
O5	−0.3572 (10)	0.6136 (4)	0.26831 (16)	0.1106 (14)	
Cl1	0.2654 (4)	0.36516 (11)	0.19545 (7)	0.1142 (6)	

### Atomic displacement parameters (Å^2^)

**Table:**

	*U*^11^	*U*^22^	*U*^33^	*U*^12^	*U*^13^	*U*^23^
C1	0.0353 (16)	0.0357 (16)	0.0387 (16)	−0.0019 (13)	−0.0005 (13)	0.0035 (13)
C2	0.0455 (18)	0.0432 (18)	0.0428 (18)	−0.0068 (14)	0.0034 (14)	−0.0003 (14)
C3	0.066 (2)	0.047 (2)	0.0379 (17)	−0.0164 (18)	−0.0092 (16)	0.0070 (15)
C4	0.081 (3)	0.0361 (18)	0.055 (2)	−0.0019 (18)	−0.020 (2)	0.0122 (16)
C5	0.065 (2)	0.0406 (19)	0.061 (2)	0.0128 (17)	−0.0061 (18)	−0.0025 (17)
C6	0.0497 (19)	0.0431 (18)	0.0426 (18)	0.0047 (15)	0.0023 (14)	0.0036 (15)
C7	0.0315 (15)	0.0367 (16)	0.0382 (16)	0.0010 (12)	0.0023 (12)	0.0074 (13)
C8	0.0339 (16)	0.0375 (17)	0.058 (2)	0.0063 (13)	0.0102 (14)	0.0103 (15)
C9	0.0362 (16)	0.0367 (17)	0.057 (2)	0.0063 (13)	0.0090 (15)	0.0100 (15)
C10	0.0334 (16)	0.0382 (17)	0.0453 (18)	−0.0002 (13)	0.0020 (13)	0.0058 (14)
N1	0.0332 (13)	0.0367 (14)	0.0585 (17)	0.0061 (11)	0.0110 (12)	0.0114 (13)
N2	0.086 (3)	0.072 (2)	0.055 (2)	−0.028 (2)	−0.0038 (19)	0.0273 (19)
O1	0.0343 (12)	0.0480 (14)	0.0831 (18)	0.0081 (10)	0.0141 (11)	0.0201 (13)
O2	0.0425 (13)	0.0464 (14)	0.0786 (18)	0.0124 (10)	0.0180 (12)	0.0196 (12)
O3	0.0525 (15)	0.0407 (14)	0.0837 (19)	0.0100 (11)	0.0238 (13)	0.0218 (13)
O4	0.126 (3)	0.192 (5)	0.098 (3)	−0.007 (3)	0.001 (2)	0.094 (3)
O5	0.144 (4)	0.111 (3)	0.079 (2)	−0.002 (3)	0.042 (2)	0.026 (2)
Cl1	0.1907 (17)	0.0468 (6)	0.1045 (11)	0.0161 (8)	−0.0287 (10)	0.0271 (6)

### Geometric parameters (Å, º)

**Table:**

C1—C2	1.381 (4)	C7—C8	1.511 (4)
C1—C6	1.384 (4)	C8—C9	1.503 (4)
C1—N1	1.411 (4)	C8—H8A	0.9700
C2—C3	1.378 (5)	C8—H8B	0.9700
C2—H2	0.9300	C9—C10	1.493 (4)
C3—C4	1.381 (6)	C9—H9A	0.9700
C3—N2	1.470 (5)	C9—H9B	0.9700
C4—C5	1.373 (5)	C10—O2	1.238 (4)
C4—Cl1	1.729 (4)	C10—O3	1.275 (4)
C5—C6	1.381 (5)	N1—H1N	0.847 (18)
C5—H5	0.9300	N2—O4	1.198 (5)
C6—H6	0.9300	N2—O5	1.213 (5)
C7—O1	1.216 (3)	O3—H3O	0.834 (19)
C7—N1	1.348 (4)		
			
C2—C1—C6	119.3 (3)	C9—C8—H8A	109.3
C2—C1—N1	118.4 (3)	C7—C8—H8A	109.3
C6—C1—N1	122.2 (3)	C9—C8—H8B	109.3
C3—C2—C1	120.1 (3)	C7—C8—H8B	109.3
C3—C2—H2	119.9	H8A—C8—H8B	107.9
C1—C2—H2	119.9	C10—C9—C8	115.2 (3)
C2—C3—C4	121.1 (3)	C10—C9—H9A	108.5
C2—C3—N2	115.9 (4)	C8—C9—H9A	108.5
C4—C3—N2	122.9 (3)	C10—C9—H9B	108.5
C5—C4—C3	118.3 (3)	C8—C9—H9B	108.5
C5—C4—Cl1	117.3 (3)	H9A—C9—H9B	107.5
C3—C4—Cl1	124.3 (3)	O2—C10—O3	122.9 (3)
C4—C5—C6	121.5 (4)	O2—C10—C9	121.8 (3)
C4—C5—H5	119.2	O3—C10—C9	115.3 (3)
C6—C5—H5	119.2	C7—N1—C1	126.4 (3)
C5—C6—C1	119.6 (3)	C7—N1—H1N	118 (2)
C5—C6—H6	120.2	C1—N1—H1N	116 (2)
C1—C6—H6	120.2	O4—N2—O5	122.2 (4)
O1—C7—N1	122.9 (3)	O4—N2—C3	119.5 (5)
O1—C7—C8	122.5 (3)	O5—N2—C3	118.3 (4)
N1—C7—C8	114.6 (2)	C10—O3—H3O	117 (3)
C9—C8—C7	111.7 (2)		
			
C6—C1—C2—C3	0.6 (5)	O1—C7—C8—C9	2.6 (5)
N1—C1—C2—C3	179.1 (3)	N1—C7—C8—C9	−176.4 (3)
C1—C2—C3—C4	−1.5 (5)	C7—C8—C9—C10	178.9 (3)
C1—C2—C3—N2	175.0 (3)	C8—C9—C10—O2	10.1 (5)
C2—C3—C4—C5	1.1 (5)	C8—C9—C10—O3	−170.8 (3)
N2—C3—C4—C5	−175.1 (3)	O1—C7—N1—C1	3.8 (5)
C2—C3—C4—Cl1	−175.8 (3)	C8—C7—N1—C1	−177.2 (3)
N2—C3—C4—Cl1	8.1 (5)	C2—C1—N1—C7	147.1 (3)
C3—C4—C5—C6	0.1 (6)	C6—C1—N1—C7	−34.5 (5)
Cl1—C4—C5—C6	177.2 (3)	C2—C3—N2—O4	−151.7 (4)
C4—C5—C6—C1	−1.0 (5)	C4—C3—N2—O4	24.7 (6)
C2—C1—C6—C5	0.6 (5)	C2—C3—N2—O5	28.7 (5)
N1—C1—C6—C5	−177.8 (3)	C4—C3—N2—O5	−155.0 (4)

### Hydrogen-bond geometry (Å, º)

**Table:**

*D*—H···*A*	*D*—H	H···*A*	*D*···*A*	*D*—H···*A*
N1—H1*N*···O1^i^	0.85 (2)	2.28 (3)	3.006 (3)	144 (3)
O3—H3*O*···O2^ii^	0.83 (2)	1.84 (2)	2.667 (3)	176 (4)

Symmetry codes: (i) *x*−1, *y*, *z*; (ii) −*x*, −*y*+3, −*z*.
